# Epithelial Cells as a Transmitter of Signals From Commensal Bacteria and Host Immune Cells

**DOI:** 10.3389/fimmu.2019.02057

**Published:** 2019-08-28

**Authors:** Yoshiyuki Goto

**Affiliations:** ^1^Division of Molecular Immunology, Medical Mycology Research Center, Chiba University, Chiba, Japan; ^2^Division of Mucosal Symbiosis, International Research and Development Center for Mucosal Vaccines, The Institute of Medical Science, The University of Tokyo, Tokyo, Japan

**Keywords:** commensal bacteria, intestinal epithelial cells, immune system, out-side in signal, inside-out signal

## Abstract

Intestinal epithelial cells (IECs) are non-hematopoietic cells that form a physical barrier against external antigens. Recent studies indicate that IECs have pleiotropic functions in the regulation of luminal microbiota and the host immune system. IECs produce various immune modulatory cytokines and chemokines in response to commensal bacteria and contribute to developing the intestinal immune system. In contrast, IECs receive cytokine signals from immune cells and produce various immunological factors against luminal bacteria. This bidirectional function of IECs is critical to regulate homeostasis of microbiota and the host immune system. Disruption of the epithelial barrier leads to detrimental host diseases such as inflammatory bowel disease, colonic cancer, and pathogenic infection. This review provides an overview of the functions and physiology of IECs and highlights their bidirectional functions against luminal bacteria and immune cells, which contribute to maintaining gut homeostasis.

## Introduction

The gastrointestinal (GI) tract is derived from progenitors in the foregut endoderm. It is a mucosal tissue covered by mucus and the major site for digestion and absorption of nutrients and water. The GI tract is therefore a unique organ that is constitutively exposed to various foreign antigens. Intestinal epithelial cells (IECs) covering the GI tract are non-hematopoietic cells that have a role in anatomical segregation of these innumerable luminal antigens such as food-derived materials and commensal microorganisms, as well as pathogens from host intestinal tissues (1). Because IECs form a protective wall against luminal antigens, the epithelial barrier is critical to maintain homeostasis of the GI tract. Indeed, disruption of the epithelial barrier by genotoxic irritants and endogenous genetic dysfunction leads to abnormal infiltration of luminal antigens and the development of inflammatory bowel diseases (IBDs) and infectious diseases (2).

In addition to the physical barrier function, IECs produce immunological molecules, such as mucus, antimicrobial molecules, and carbohydrate moieties, as well as secretory immunoglobulin A (SIgA) derived from plasma cells into the lumen (3). These chemical modulators prevent aberrant attachment and infiltration of luminal antigens into intestinal tissues. IECs also produce cytokines and chemokines as well as hormones that serve as modulators to fine tune the immune and nervous systems in the gut (3, 4). Therefore, IECs function as a bidirectional modulator of luminal antigens and the host.

Important environmental factors that modulate host metabolic and immune homeostasis are commensal microorganisms including bacteria, fungi, parasites, and viruses (5). In particular, commensal bacteria are well-recognized as a booster of mucosal immune responses such as IgA production, T cell development and activation, and production of antimicrobial peptides by IECs (6, 7). Perturbation of commensal microbiota, which is termed as dysbiosis, and subsequent abnormal immune responses are pre-dispositions for the development of a range of local and systemic diseases such as IBDs, obesity, diabetes, cancer, and even autism (6). Recent studies have shown that IECs are important to recognize stimulation by commensal bacteria and direct induction and regulation of immune responses in the intestines. In contrast, immune cells underlining IECs direct production of immunological factors that influence luminal microbiota (8, 9). In this context, IECs coordinate crosstalk between luminal commensal bacteria and gut immune cells. This review highlights recent advances in understanding the unique features of IECs accompanied by luminal microbes and adjacent immune cells, and discusses unique functions of IECs as a bidirectional modulator of luminal bacteria and the immune system to maintain gut homeostasis.

## Epithelial Subsets and Phenotypes in the Intestines

The small intestine has unique protrusion structures called villi that contribute to extending the mucosal surface area to absorbing nutrients. In contrast, colon defects of villi have a relatively flat structure to prevent potential damage caused by intestinal contents in the event of transition from the upper to lower portion. IECs covering the intestinal tract are morphologically and functionally heterogeneous and have pleiotropic roles as a surface barrier system. Each epithelial cell subset, including enterocytes, goblet cells, Paneth cells, tuft cells, enteroendocrine cells, microfold (M) cells, and epithelial stem cells, have unique and specialized gene expression and functions, which cooperatively form a sophisticated epithelial layer against numerous antigens in the lumen. Disruption of the epithelial barrier allows abnormal intrusion of luminal antigens, including commensal bacteria, which causes detrimental inflammatory diseases such as IBDs, infection, and sepsis.

Epithelial cells (ECs) in the intestine are categorized into various subsets. The most prominent type of ECs are enterocytes that constitutively form tight junction structures and create the first line of the physical barrier against luminal antigens at the mucosal surface (1). In addition to physical barrier formation, enterocytes have the potential to produce antimicrobial molecules, such as regenerating islet-derived 3 (RegIII) γ and Ly6/PLAUR domain containing 8 (Lypd8), and generate various carbohydrate chains (8, 10, 11). In the context of immunological functions, enterocytes express polymeric immunoglobulin receptor (pIgR) at the basolateral side of the cell membrane. After binding to pIgR, dimeric IgA produced by lamina propria plasma cells transcytoses to the apical surface and is subsequently released as SIgA into the lumen.

Goblet cells are well-characterized as mucus-producing cells. The epithelial layer is covered by two mucus layers in the intestines, specifically thick inner and thin outer mucus layers (12). Mucin encoded by the Muc2 gene is a major component of intestinal mucus. Because deletion of the Muc2 gene leads to a defect of the inner mucus layer, commensal microbes can access the epithelial surface. The constitutive and abnormal bacterial stimulation observed in Muc2-deficient mice induces pathological inflammation and tumorigenesis in the colon (13, 14). Recently, sentinel goblet cells have been characterized in the colon. These cells produce mucin in response to bacterial signals, and NOD-like family pyrin domain containing 6 (NLRP6)/inflammasomes govern exocytosis of mucin into the lumen (15). Goblet cells also produce anti-inflammatory molecules such as trefoil factor 3, a tissue-protective factor that repairs the epithelial layer by inhibiting apoptosis, and resistin-like molecule (RELM) β (16, 17).

Paneth cells reside in the crypt basal region and intercalate with epithelial stem cells. They are specialized to produce anti-microbial molecules such as lysozyme, α-defensins, secretory phospholipase A2 (sPLA2), and RegIIIγ (11, 18). Paneth cells also express epidermal growth factor (EGF), Wnt3, and the Notch ligand delta-like 4 (Dll4) to maintain homeostasis of epithelial stem cells (19). Therefore, disruption of Paneth cell functions caused by aberrant endoplasmic reticulum stress and autophagy signals leads to pathogenic infection and IBDs (20, 21). Because Paneth cells are observed in the small intestine, but not in the colon, the anatomical location of typical epithelial cell subsets governs intestinal functions. Antimicrobial peptides specifically expressed in the colon may compensate for the function of Paneth cells in the small intestine (10). A recent single cell study of intestinal epithelial cell subsets provided evidence that each subset is functionally heterogeneous and ECs isolated from distinct intestinal regions have different phenotypes even in the same subsets (22). For example, Paneth cells, a secretor of antibacterial molecules, can be classified into two types: Paneth-1 and-2. Paneth-1 cells, which highly express α-defensin, are abundant in the ileum. In contrast, Paneth-2 cells, which preferentially express RNase1, are enriched in the duodenum. Although it is unclear why there is such a regional distribution of the subsets of each IEC type, several reports suggest that region-specific environmental stimuli may affect epithelial physiology in the gut. In particular, specific commensal bacteria colonizing a specific region of the intestinal tract direct IECs to express specific antibacterial peptides, carbohydrate moieties, and immune modulatory molecules that induce immune responses *in situ*.

Another unique epithelial subset—Tuft cells—sense luminal helminths, such as *Tritrichomonas muris*, through their GTP-binding protein, α-gustducin (23). Tuft cells produce IL-25 and thymic stromal lymphopoietin (TSLP) in response to helminths to induce Th2-type immune responses and protect against infection by a helminth (24).

M cells reside in the follicle-associated epithelium (FAE) covering secondary lymphoid tissues such as Peyer's patch and isolated lymphoid follicles. They have unique morphological characteristics such as irregular microvilli and pocket structures containing lymphocytes and dendritic cells (DCs). M cells take up antigens from the lumen as well as serve as an entrance for pathogenic and non-pathogenic microorganisms. A recent report showed that allograft inflammatory factor 1 (Aif1) is a critical molecule for uptake of antigens such as those of *Salmonella typhimurium* (25). Receptor activator of nuclear factor-κB ligand (RANKL) and TNF receptor-associated factor 6 (TRAF6)-mediated NF-κB signaling regulate differentiation of M cells. Mesenchymal cells residing under the FAE produce RANKL that is critical for the differentiation of M cells via epithelial RANK (26, 27). All IECs derived from stem cells expressing leucine-rich repeat-containing G protein-coupled receptor 5 (Lgr5) reside at the base of the crypt. These stem cells become transient proliferative cells that differentiate into each mature epithelial subset.

## Microbiota Directly Affects Epithelial Physiology

More than 1 × 10^13^ bacteria symbiotically colonize the human intestines (28). Each bacterial species adapts to the specific intestinal environment for colonization, such as oxygen concentration, pH, redox potential, nutrient supplies, host secretions, and intestinal motility. Therefore, each bacterial species colonize at different sections along the intestines (29, 30). Mice are usually maintained under specific pathogen-free (SPF) conditions with sterile chow and water. Even under this condition, inter-mouse variations are observed from the phylum to the operational taxonomic unit (OTU) levels, especially in gastric and small intestinal samples. Fecal microbiota has a relatively similar bacterial population as the large intestine (30). A community of bacteria resides in each GI tract region of wildtype (WT) mice. At the family level, anaerobes including *Bacteroidaceae, Prevotellaceae, Rikenellaceae, Lachnospiraceae*, and *Ruminococcaceae* were enriched in the large intestine and feces, while *Lactobacillaceae* predominantly colonized the small intestine and stomach in a murine model (30). At the genus level, the large intestine and feces had a higher percentage of *Bacteroides, Prevotella*, and *Alistipes*, while *Lactobacillus* had a higher proportion in the stomach and small intestine (30). In the stomach and upper part of the small intestine, the number of bacteria is low compared with the lower part of the GI tract (31). It is difficult to discriminate “transient” bacteria that pass through the intestines and “colonized” bacteria that proliferate and are stably observed in the intestines. Mice usually feed on feces that may pass through the stomach and small intestine, which may also affect the bacterial population.

Importantly, the functions of IECs are affected by stimulation of luminal antigens including commensal bacteria. As reported previously, commensal bacteria influence epithelial physiology and subsequent intestinal lymphoid structures and can cause abnormal villous morphology and epithelial cell proliferation (32). The mucus layer is thick in the distal colon in concert with the abundance of goblet cells. This gradient is parallel to the load of commensal bacteria. It has been reported that Toll-like receptor (TLR) and NLRP6 signaling in IECs control differentiation of goblet cells in response to microbial stimulation (33, 34). Therefore, germ-free (GF) mice and antibiotic-treated mice have a thin mucus layer. Production of antimicrobial peptides such as RegIIIγ in GF mice diminishes compared with WT mice (35). In addition, replication of epithelial stem cells is disrupted, and thus, antibiotic-treated mice are susceptible to colitis induced by physical and chemical disruption of IECs (36). TLR2, TLR4, and Myd88, a downstream signaling molecule of these receptors, are responsible for detection of commensal bacteria. Epithelial cell proliferation is abnormal in mice lacking TLR2, TLR4, and Myd88, and these mice are susceptible to dextran sulfate sodium (DSS)-induced colitis (36). Furthermore, TLR-commensal bacterium interactions are important to form an epithelial barrier by maintaining tight junction proteins in IECs (36, 37). Deficiency of such TLR signaling in IECs exacerbates colitis induced by pathogenic bacteria such as *Citrobacter rodentium* (38).

Intestinal bacteria synthesize a variety of materials derived from foods as well as carbohydrates secreted from host IECs as a consequence of their metabolism. These metabolites have been reported to stimulate IECs and modulate epithelial physiology. Important metabolites produced by commensal bacteria are short chain fatty acids (SCFAs) such as acetate, propionate, and butyrate. These SCFAs are usually generated from the process of fermentation of dietary fibers. *Bifidobacterium longum*, an obligate anaerobe, produces acetate that protects IECs against apoptosis induced by O157 toxin (39). Acetate also induces goblet cell differentiation, secretion of mucin, and decoration of mucin glycans with sialic acid (40). Because of their lack of commensal bacteria, GF mice have shorter Muc2 O-glycans and express several glycosyltransferases at a low level in IECs (40). Other anaerobic bacteria, such as *Clostridium* clusters IV and IXa, *Faecalibacterium prausnitzii*, and *Bacteroides thetaiotaomicron*, produce butyrate. Although butyrate is an important SCFA to establish the gut immune system, as discussed later, butyrate represses the proliferation of intestinal stem cells during DSS treatment (41). This may prevent abnormal transformation of IECs under exogenous stress from the luminal environment. Butyrate diminishes proliferative activity by inhibiting histone deacetylase (HDAC) enzymes and inducing Forkhead box O3 (Foxo3) expression in epithelial stem cells (41). The colonic crypt architecture is suggested to protect against exposure of a high concentration of butyrate to these epithelial stem cells (41). Furthermore, butyrate from commensal bacteria promotes colonic oxygen consumption by stabilizing the transcription factor Hypoxia-inducible factor (HIF), which augments epithelial barrier functions (42).

In addition to SCFAs, other metabolites from bacteria such as lactate induce hyperproliferation of colonic ECs (43). Because lactate is an energy source, epithelial stem cells use lactate produced by adjacent Paneth cells, which promotes differentiation and proliferation (44). Despite these accumulating data, it is still controversial whether lactate-producing bacteria such as *Lactobacillus* influence epithelial stem cell homeostasis *in vivo*, especially in humans. Taken together, commensal bacteria and commensal-derived metabolites are important to maintain homeostasis of the epithelial barrier. Dysbiosis observed in IBD patients and colitis mouse models, such as an expansion of *Enterobacteriaceae*, may presumably reduce these metabolites, leading to disruption of the epithelial barrier system (45, 46).

In the intestines, fungi and bacteria compete for niches, and these microbes influence each other. Although the number of fungi is relatively low compared with bacteria, fungi are a major microbial population in the gut. For example, *Candida, Saccharomyces, Aspergillus, Cryptococcus, Malassezia, Cladosporium, Galactomyces*, and *Trichosporon* have been reported to colonize human intestines (47–49). In contrast to humans, fungi colonization in the intestines of experimental mice is dependent on the animal facility. Whereas, fungi are observed in the gut of WT mice, termed as mycobiota, laboratory mice bred in some facilities are resistant to fungal colonization in the steady state. This could be because the composition of commensal bacteria colonizing mice is distinct between animal facilities. Indeed, depletion of gut microbiota by antibiotic treatment allows colonization of *Candida* and *Saccharomyces* in the gut (50, 51). Among commensal microbiota, *Blautia producta* and *B. thetaiotamicron* have been identified as the bacteria responsible for colonization resistance against *C. albicans*. These bacteria induce production of cathelicidin-related antimicrobial peptide (CRAMP) from IECs, which is mediated by the transcription factor hypoxia-inducible factor-1α, resulting in the inhibition of colonization of the gut by *C. albicans* (50). Interestingly, colonization of *Candida* and *Saccharomyces* ameliorates DSS-induced colitis, although the detailed mechanism is still unclear (51). Therefore, luminal microorganisms, including bacteria and fungi, are closely associated with the intestinal epithelial physiology.

## “Outside-in Signals” From Microbiota Modulate Immune Responses

A difficulty of research related to commensal bacteria is that the environment of the animal facility, especially food and water, affects commensal microbiota. For example, the numbers of segmented filamentous bacteria (SFB) and *Lactobacillus murinus* are dramatically reduced in C57BL/6 mice bred in Jackson laboratory, but not in Taconic farm, which is closely associated with the development of host mucosal immune systems such as the development of T helper 17 (Th17) cells (35). Antibiotic-treated and GF mice are widely used to investigate the effect of commensal bacteria on host physiological and pathological functions. In addition, researchers have established GF mice colonized with specific bacteria, termed gnotobiotic mice, using a vinyl isolator under sterile conditions to uncover the role of each bacterial species. It has been reported that colonization of specific bacteria triggers the development of immune cells in the gut (6). The intestinal immune system has tolerance for commensal bacteria. However, this tolerance does not mean no response, but a response to commensal bacteria without pathogenic symptoms, which is also considered as “physiological inflammation.” Indeed, the numbers of T cells and IgA^+^ cells are strikingly reduced and secondary lymphoid organs are immature in GF mice compared with WT mice. Whereas, commensal bacteria directly stimulate immune cells in lamina propria in some cases, IECs stimulated by commensal bacteria also initiate immune responses. This review terms this cascade from commensal bacteria to immune cells through IECs as “outside-in signals.” IECs recognize signals from various commensal species and transmit these outside-in signals to immune cells. Therefore, IECs play a central role in the establishment of the gut immune system in response to commensal bacteria.

Intestinal epithelial cells (IECs) recognize luminal bacterial signals by a variety of pattern recognition receptors including TLRs and nod-like receptors (NLRs). Enterocytes express TLR2, TLR3, TLR4, TLR5, and TLR9 (52, 53). Unlike immune cells, IECs have the unique feature of cellular polarization that facilitates the anatomical distribution of TLRs. Although IECs usually express TLRs at the basolateral membrane to circumvent the induction of detrimental inflammatory responses, TLR2 and TLR9 are also expressed on the apical side of IECs (54). TLR signaling in IECs leads to the expression of inflammatory cytokines and chemokines such as IL1β, IL-6, IL-18, and CCL20. In contrast, apical stimulation of TLR9 induces an immune inhibitory effect through stabilization of IκB, demonstrating the unique ability of IECs to differentially respond to microbial signals using the same receptors expressed at apical and basolateral positions (55). In addition to TLRs, IECs express NLRs to detect bacterial components and danger signals. The expression and functions of NLR in IECs are summarized in another review (56).

**Figure 1:**
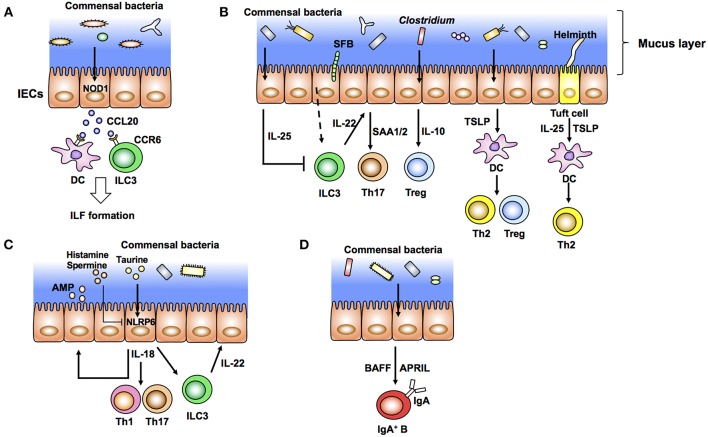
Intestinal epithelial cells (IECs) modulate the gut immune system in response to commensal bacteria (outside-in signals). **(A)** Diverse microbiota provides the ligands of NOD1 expressed in IECs. These ligands induce production of epithelial CCL20 as well as recruitment of CCR6^+^ dendritic cells (DCs) and group 3 innate lymphoid cells (ILC3) that initiate the development of isolated lymphoid follicles (ILFs). **(B)** Specific commensal bacteria, such as segmented filamentous bacteria (SFB) and *Clostridium*, induce differentiation of Th17 cells and Tregs, respectively. SFB induce production of IL-22 from ILC3. IL-22 elicits epithelial SAA1/2 and subsequent Th17 cell differentiation. IECs produce IL-10 in response to Clostridia and induce Treg differentiation. Epithelial IL-25 limits the production of IL-22 from ILC3. IECs condition dendritic cells (DCs) to a tolerogenic phenotype through the production of TSLP. Tuft cells recognize helminth signals and produce IL-25 and TSLP that skew Th2 immune responses. **(C)** Epithelial IL-18 production is mediated by activation of NLRP6 in response to taurine produced by commensal bacteria. Epithelial IL-18 induces the production of IL-22 from ILC3 and antimicrobial peptides (AMP) from epithelial cells in an autocrine manner. **(D)** Commensal bacteria elicit production of APRIL and BAFF by IECs and IgA class switching of B cells.

Commensal bacteria induce development and maturation of secondary lymphoid organs such as Peyer's patch and isolated lymphoid follicles (ILFs). Indeed, Peyer's patches are hypoplastic and ILFs are hardly observed in GF and antibiotic-treated mice. The FAE covering Peyer's patch and ILFs recognizes luminal bacteria and initiates and organizes these secondary lymphoid organs. Antigen-presenting cells located under FAEs are mostly DCs. These DCs take up luminal antigens from M cells in FAEs and prime T cells and subsequent activation of B cells to initiate antigen-specific immune responses. FAE induces structural organization of Peyer's patch and ILFs by producing chemokines such as CCL20 and CCL9 in mice and CCL20 and CCL23 in humans (57, 58). DCs localized in the subepithelial dome region are recruited by chemokines such as macrophage inflammatory protein (MIP) 3α (59). CCL20 expressed by FAEs also recruits CCR6^+^ B cells into ILFs and Peyer's patch follicles (60). Although the bacterial recognition system in FAE of Peyer's patch is still mostly unclear, a set of TLRs expressed in the FAE may affect such chemokine expression (61). In the context of ILF development, nucleotide-binding oligomerization domain containing 1 (NOD1) expressed in FAEs recognizes peptidoglycans (PGNs) derived from Gram-negative bacteria and induces CCL20 expression and subsequent recruitment of CCR6^+^ DCs and group 3 innate lymphoid cells (ILC3) (62) (Figure 1A).

Intraepithelial lymphocytes (IELs) are a T cell subset spatially distributed between IECs. Homeostasis of IELs, especially TCRαβ, but not TCRγδ IELs, is maintained by signals from commensal bacteria. In GF mice, the number of IELs is dramatically reduced compared with WT mice (63). Several reports have shown that specific commensal bacterial species are involved in the development of IELs. For example, SFB and *Lactobacillus reuteri* induce IEL subsets such as CD4^+^ CD8αα^+^ IELs (63, 64). Although metabolites produced by commensal bacteria directly affect IEL development, IECs also mediate IEL development and functions. IL-15 expressed by IECs participates in IEL maintenance through trans-presentation of IL-15 to IL-15Ra expressed on IELs (65). Although the mechanism is still unknown, IL-15 is induced following exposure to commensal bacteria (66). In addition, secretion of IL-15 by IECs is dependent on MyD88. Therefore, the numbers of IELs are reduced in MyD88-deficient mice, which can be restored upon transgenic expression of IL-15 (67). This suggests that commensal signals regulate IEL numbers through induction of IL-15 production by IECs.

In the lamina propria region, differentiation of T helper cells, especially Th1, Th17, Tregs, and CD8 T cells, is controlled by various commensal microbes (35, 46, 68–72) (Figure 1B). Among these T cells, IL-17-producing Th17 cells positive for the transcription factor RORγt are induced by SFB and a mixture of 20 species of human commensal bacteria (35, 72) (Figure 1A). SFB specifically colonize at the epithelial layer in the ileum and are closely associated with IECs. This association between bacteria and IECs is critical to induce Th17 cells (71). Rat-derived SFB, which are not able to attach to murine IECs, do not induce Th17 cells in mice (71). In addition, *C. rodentium* expressing structural protein intimin interact with IECs and induce Th17 cells. However, Th17 cells are not induced in mice infected with an intimin-deficient strain (71). In addition to bacteria, pathogenic fungus *C. albicans* colonize the epithelial surface and induce Th17 cells (71). Epithelium-associated *Escherichia coli* and *Bifidobacteria adolescentis* isolated from humans also induce inflammatory Th17 cells (73). In addition to these pathogenic and non-pathogenic microorganisms, *Acinetobacte*r spp., *Bacteroides fragilis*, and Proteobacteria have been reported as epithelium-associated bacteria (74). However, it is still unknown whether these bacteria have the potential to induce Th17 cells.

In the process of Th17 cell development, production of IL-22 by ILC3 in response to SFB attachment to microvilli of IECs elicits serum amyloid A proteins 1 and 2 (SAA1/2) from ileal ECs, which augment Th17 cell development (75) (Figure 1A). Importantly, IL-22 produced by ILC3 trigger activation of signal transducer and activator of transcription 3 (STAT3), a transcription factor downstream of the IL-22 receptor, in IECs. SAAs derived from ileal ECs are critical for the development of Th17 cells (75). Although the mechanism of how the association of SFB with IECs induces IL-22 production by ILC3 is still unclear, reports suggest that IECs have a role in the initiation and enhancement of Th17 cell development. Th17 cells and IL-22 production by ILC3 are important to prevent infection by *C. rodentium* (35). In addition to bacteria, *T. musculis*, a previously unrecognized commensal protozoan, activates NLRP6 in IECs and subsequent IL-18 production. Microbiota-derived metabolites, such as taurine, histamine, and spermine, regulate secretion of IL-18 and anti-microbial peptides by modulating NLRP6 inflammasome signaling (15) (Figure 1C). Taurine derived from microbiota enhances IL-18 production in IECs (Figure 1C). In contrast, histamine and spermine inhibit this IL-18 production (15) (Figure 1C). Defects in epithelial NLRP6 reduce cleavage of caspase-1 and subsequent IL-18 secretion as well as the expression of antimicrobial peptides that predispose to the development of dysbiosis and colonic inflammation (15, 76). These microbiota-derived metabolites, epithelial IL-18 production, and the anti-microbial peptide production cascade are important to maintain microbiota diversity and colonic homeostasis. Indeed, NLRP6-deficient mice have spontaneous intestinal hyperplasia, inflammatory cell recruitment, and exacerbated experimental colitis caused by pathobiont colonization (76). In contrast to this report, other groups have recently shown that NLRP6 deficiency has no effect on the community of gut microbiota (77, 78). These reports indicate the importance of performing experiments with littermates when addressing the function of gut microbiota. Several factors including mouse facilities and experimental designs may also account for the discrepancies in the results. Therefore, the role of NLRP6 in IECs against microbiota is still controversial. Epithelial IL-18 also enhances the production of IL-22 from ILC3 in the steady state (79) (Figure 1C). Because IL-18 secreted from IECs augments inflammatory Th1 and Th17 cell differentiation, mice infected with *T. musculis* are susceptible to T cell-induced colitis and sporadic colorectal tumors compared with uninfected mice (80, 81). In contrast, *T. musculis*-infected mice are resistant to mucosal infection by *S. typhimurium* (80). These studies indicate that IECs modulate intestinal pathogenesis as well as homeostasis in response to commensal bacteria.

In addition to the development of Th17 cells, IECs coordinate anti-inflammatory Th cell responses. *Clostridium* class IV, class XIVa, and cluster XVIII and *B. fragilis* have been reported to induce Tregs in the colon (68, 69, 71) (Figure 1B). In particular, colonization of *Clostridium* elicits production of IL-10, a potent anti-inflammatory cytokine, from IECs, which enhances differentiation of colonic Tregs (68) (Figure 1B). IECs also direct anti-inflammatory Treg and Th2 cell differentiation by producing TSLP after bacterial stimulation (Figure 1B). Expression of TSLP is elevated in IECs treated with non-invasive bacteria *in vitro* (82). TSLP from IECs conditions DCs to an anti-inflammatory status and promotes polarization of T cells toward Tregs and Th2 cells (82) (Figure 1B). TSLP derived from IECs prevents IL-12-stimulated induction of DCs and enhances the production of IL-6 and IL-10. Expression of IL-12, a Th1-prone cytokine, from DCs is therefore augmented under a TSLPR-deficient condition (83). IEC-intrinsic IKKβ controls TSLP induction. Thus, both IKKβ- and TSLPR-deficient mice have defects in Th2 responses and are susceptible to parasitic *Trichuris* infection (83). IECs also induce anti-inflammatory Th2 cytokine IL-25 (IL-17E) in response to commensal bacteria. Thus, IL-25 expression is defective in GF mice. IL-25 from IECs inhibits IL-22 production from ILC3 (84, 85) (Figure 1B). Because IL-22 from ILC3 is important for epithelial cell proliferation, IL-25 inhibits epithelial tissue repair and exaggerates DSS-induced colitis (84). Taken together, intestinal ECs transmit signals from luminal bacteria to immune cells and trigger positive and negative T cell responses to maintain gut homeostasis.

Commensal bacteria are also important to induce intestinal immunoglobulin A (IgA). IgA production is impaired in GF mice, which is correlated with immature secondary lymphoid tissues (32). The IgA induction by commensal bacteria may depend on anatomical colonization of specific commensal bacteria. For example, SFB induce IgA in the ileum, but a mixture of 46 *Clostridia* and *Bacteroides acidifaciens* induce IgA in the colon (86, 87). In addition to SFB, epithelium-associated commensal bacteria, such as *Mucispirillum*, activate T cell-dependent IgA production (88). In the lamina propria region, IgA is induced in a T cell-independent manner (89). In this context, IEC-derived cytokines, especially TNF superfamily members, B cell-activating factor of the tumor necrosis factor family (BAFF), and proliferation-inducing ligand (APRIL), induce class switching to IgA2, the main mucosal IgA class in humans (Figure 1D). TSLP produced from IECs triggers APRIL production by DCs in response to TLR-mediated signals from commensal bacteria (90). Ectopic expression of TLR4 in IECs augments the expression of CCL20, CCL28, and APRIL that recruit and activate lamina propria (LP) CCR6^+^ B cells and IgA class switching (91). These data support a model in which IECs stimulated by commensal bacteria initiate T cell-independent IgA production.

## “Inside-out Signals” Modulate Microbiota

Because of the development of experimental tools to analyze bacterial DNA, such as next-generation sequencing with bioinformatics and quantitative PCR, the number, diversity, population, and gene expression of the microbiota have been investigated comprehensively. Using these powerful tools, it is possible to analyze microbiota isolated from specific patients and genetically modified murine models. Based on the accumulated studies, it has been reported that intestinal immune cells together with IECs affect luminal microbiota. Because of the anatomical features of IECs exposed to the luminal environment, immunological factors are usually produced by IECs. However, intestinal immune cells express various bacterial recognition receptors. Therefore, immune cells underlying IECs detect bacterial signals and transfer the signal to IECs to produce immunological factors. The cascade from immune cells to commensal bacteria through IECs is termed as “inside-out signals” in this review. In addition to signals from the luminal environment, resident immune cells adjacent to IECs calibrate epithelial physiology and luminal microbiota.

**Figure 2:**
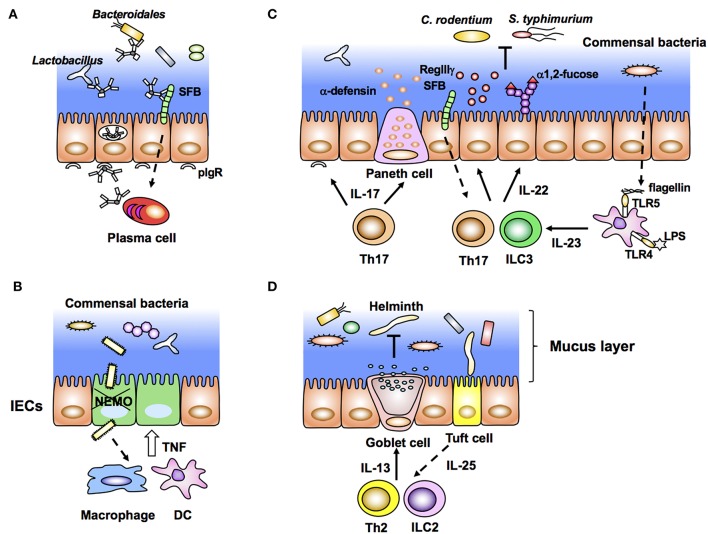
Intestinal epithelial cells (IECs) stimulated by immune cells affect gut microbiota (inside-out signals). **(A)** Dimeric IgA antibodies produced by plasma cells in the lamina propria bind to pIgR expressed on the basolateral membrane of IECs, undergo transcytosis, and are secreted into the lumen as SigA. SIgA binds to commensal bacteria and maintains their homeostasis. **(B)** NEMO deficiency in IECs allows bacterial infiltration that leads to aberrant production of TNF from macrophages/DCs and further apoptosis of IECs. **(C)** Lamina propria DCs produce IL-23 in response to bacterial flagellin and LPS. IL-23 induces production of IL-22 from group 3 innate lymphoid cells (ILC3) and Th17 cells, leading to expression of epithelial anti-microbial molecules, such as RegIIIγ and α1,2-fucose. RegIIIγ and α1,2-fucose, which regulate the luminal microbial population. **(D)** Tuft cells produce IL-25 in response to helminth infection. Epithelial IL-25 promotes IL-13 production from ILC2 and Th2 cells, and subsequent production of mucus from goblet cells.

Dimeric IgA produced by plasma cells in the LP binds to pIgR expressed on the basolateral side of IECs. After binding to pIgR, IgA is transcytosed to the apical surface and subsequently released as SIgA into the lumen (Figure 2A). SIgA is important to neutralize bacterial toxins, virus infection, and invasion of pathogenic and non-pathogenic bacteria into IECs. Recent elegant studies employing IgA-seq analysis have revealed that IgA-coated microbiota has the characteristic of immunogenic commensal bacteria (88, 92, 93). IgA-seq is a method that combines IgA^+^ bacterial cell sorting and 16S rRNA gene sequencing of sorted bacteria to characterize intestinal microbiota coated with IgA. Using this method, SFB, *Bacteroidales, Lactobacillus*, and unclassified *Erysipelotrichaceae* were identified to be highly coated with IgA in SPF mice (Figure 2A). Under an inflammasome-deficient condition, mice have aberrant commensal microbiota with a colitogenic feature. In these mice, the *Prevotellaceae* family and *Helicobacter sp. flexispira*, in addition to *Lactobacillus* and SFB, are coated with IgA (93). Although it is still controversial whether SIgA regulates homeostasis of commensal bacteria, activation-induced cytidine deaminase (AID)-deficient mice with defects in immunoglobulin class switch recombination (CSR) and somatic hypermutation (SHM) have aberrant microbiota and hyperplasia of germinal center B cells (94). In particular, SHM rather than CSR is important to maintain homeostasis of gut microbiota (95). SIgA also affects bacterial gene expression. SIgA downregulates the expression of capsular polysaccharide synthesis 4 (CPS4) and enhances CPS5 expression, both of which are epitopes of *B. thetaiotaomicron* for adaptation to the intestinal environment (96).

As described above, bacterial components and metabolites directly stimulate IECs and maintain homeostasis of the epithelial barrier. However, disruption of epithelial homeostasis leads to pathogenic intestinal inflammation caused by the production of proinflammatory cytokines, such as TNF, IL-22, and IL17a, by innate and acquired immune cells. For example, IEC-specific deletion of components that activate nuclear factor-κB (NF-κB), especially the inhibitor of NF-κB (IκB) kinase (IKK) complex and NF-κB essential modulator (NEMO), results in spontaneous colitis caused by elevation of epithelial apoptosis and exaggerated DSS-induced colitis (97, 98). Commensal bacteria stimulate colonic DCs and/or macrophages through MyD88 signaling and induce aberrant TNF production, accelerating epithelial apoptosis, and inflammation (97) (Figure 2B). This inflammation is impaired in TNFRI-deficient IECs (97). These data indicate that epithelial NF-κB signals contribute to maintaining homeostasis of the epithelial barrier and inhibit excess production of inflammatory cytokines and pathogenic inflammation. In contrast to NEMO, deletion of IKKα in IECs results in TSLP overproduction and inhibition of IL-22 production by ILC3 (99). Because of the disruption of epithelial homeostasis, IKKα-deficient mice are susceptible to colitis and pathogenic infection (98).

An important and well-characterized cytokine from immune cells, which stimulates IECs is IL-22. IL-22 is a member of the IL-10 cytokine family usually produced by Th17/Th22 and ILC3 under stimulation by IL-23. Because IL-22R is constitutively and specifically expressed in IECs (100), IL-22 contributes to affecting epithelial cell proliferation, differentiation, glycosylation, and production of antimicrobial peptides. In particular, IL-22-mediated epithelial stem cell regeneration is critical to maintain gut homeostasis after genotoxic treatment and pathogenic infection (101, 102). Indeed, IL-22 induces expression of antimicrobial molecules RegIIIβ and RegIIIγ in IECs for exclusion of pathogenic bacteria such as *C. rodentium* (100) (Figure 2C). In addition to such immunological features, IL-22 production by ILC3 creates a host-commensal symbiotic platform in the gut. IECs express various carbohydrate moieties on the apical surface of their cell membrane. Fucosylated glycans are synthesized by addition of an L-fucose residue via an α1-2 linkage to the terminal β-D-galactose of glycan in a process catalyzed by fucosyltransferase (Fut), especially Fut2 expressed in IECs. This epithelial α1, 2-fucosylation is initiated by colonization of SFB and stimulation of DCs by lipopolysaccharide (LPS). After such stimulation, IL-22 from ILC3 induces Fut2 expression and α1, 2-fucosylation in IECs (8, 9) (Figure 2C). Importantly, epithelial α1, 2-fucosylation prevents infection and contributes to maintaining commensal microbiota and gut homeostasis (8, 9, 103). In contrast to the positive effect of ILC3, IL-10-producing T cells negatively regulate the induction of ectopic epithelial α1, 2-fucosylation (104). Interestingly, high numbers of α1, 2-fucose^+^ IECs and Th17 cells are observed in the ileum, but not in the duodenum (8, 105). The regional gradients of epithelial α1, 2-fucose and the number of Th17 cells correspond to the colonization of SFB. Therefore, colonization of specific microbes affects both the localization and activation/differentiation state of immune and ECs. In addition to α1, 2-fucose, expression of RegIIIγ and nitric oxide synthase 2 (NOS2), and SAA production in ileal ECs is controlled by SFB colonization (35). Although the detailed mechanism is unclear, attachment of bacteria to intestinal ECs triggers the subsequent unique development of immune cells in the gut (72). Another report has shown that IL-23 produced by CD103^+^ CD11c^+^ TLR5^+^ cells induces IL-22 from ILC3 in response to bacterial flagellin (106) (Figure 2C).

Among the members of the IL-17 cytokine family, IL-17a is mainly produced by γδ T cells and Th17 cells. IL-17a induces epithelial NF-kB signaling and maintains tight junctions (107, 108). IL17a-deficient mice are susceptible to DSS-induced colitis. Therefore, IL17a is also important to maintain the epithelial barrier function and homeostasis of the intestines (109). IL17a and IL-17R signaling in IECs elicits the expression of immunological factors, such as α-defensin, Nox1, and pIgR, which regulate SFB colonization in the intestines (110) (Figure 2C). As mentioned above, intestinal Th17 cells are induced by commensal bacteria, especially SFB, and mice colonized with SFB are resistant to infection by *C. rodentium* (35). Therefore, SFB colonization regulates homeostasis of itself and prevents infectious diseases mediated by IL-17a.

In the stomach, *Lactobacillus* produce the metabolite dietary tryptophan, a ligand of aryl hydrocarbon receptor (AHR), which also promotes differentiation of IL-22-producing ILC3. IL-22 induces secretion of antimicrobial peptides from gastric ECs, which prevent colonization by *C. albicans* (111). It has been reported that dysbiosis, which is an altered *Lactobacillus* population, is observed in caspase-associated recruitment domain 9 (CARD9)-deficient mice (112). Dysbiosis observed in mice lacking Card9 affects tryptophan metabolism and impairs stimulation of AHR, leading to impairment of IL-22 production and expression of epithelial RegIIIβ and RegIIIγ (112). In addition to the induction of IL-22, AHR activation induces epithelial cytochrome P4501 (CYP1) enzymes that oxygenate AHR ligands. The numbers of Th17 cells and ILC3 are dramatically reduced in mice with IEC-specific depletion of Cyp1a1, resulting in increased susceptibility to infection by *C. rodentium* (113).

In the context of helminth infection, the aforementioned IL-25-secreting Tuft cells condition Th2 immune responses as described above. ILC2 and Th2 cells activated by IL-25 produce IL-13 and IL-33. Notably, IL-13 enhances the production of mucus and antimicrobial peptide resistin-like molecule β (RELMβ) by goblet cells, which mediate worm expulsion (Figure 2D). In this manner, commensal bacteria, IECs, and immune cells interact with each other and create a network system at the mucosal surface, and IECs serve as key players in this interplay. The triangular regulatory machinery consisting of commensal bacteria, IECs, and immune cells is an important and representative model for understanding intestinal homeostasis (Figure 2).

## Concluding Remarks

Commensal bacteria naturally co-inhabit the intestines of their host. Because IECs encounter luminal antigens including those of these symbionts, IECs produce symbiotic factors, such as carbohydrate moieties and mucus, as well as immunological mediators including antimicrobial peptides (11, 114). In addition to SIgA, immune cells direct IECs to produce symbiotic and immunological factors, and influence the microbiota and gut homeostasis.

As discussed above, immune cells produce cytokines in response to signals from commensal bacteria. How these immune cells detect commensal bacteria is an important question that remains to be answered. One possibility is that CX3CR1^+^ cells extending their dendrites into the lumen directly detect bacterial stimulation (115). A recent study showed that lactate from commensal bacteria induces the extension of dendrites from these cells (116) and another report has shown that metabolites produced by butyrate directly stimulate T cells in the lamina propria (117). Future studies are required to identify the mechanism by which metabolites produced by bacteria in the lumen reach immune cells through IECs. Reports have shown that commensal bacteria induce Th17 cell development and IL-22^+^ ILC3 in the steady state (8, 35, 72, 75). DCs, which present bacterial antigens to T cells, are critical for the induction of Th17 cells (118, 119). In addition, IL-23 produced by TLR5^+^ DCs, which detect bacterial flagellin, induce IL-22 expression in ILC3 (106). These data suggest that bacterial antigens reach and stimulate LP DCs. How these bacterial antigens and metabolites stimulate LP immune cells and the roles of IECs in this process are still unknown. IECs may fill such an anatomical gap between commensal bacteria and immune cells. For example, goblet cells take up antigens from the lumen into LP CD11c^+^ DCs (120). Further analysis is needed to reveal the detailed mechanism by which luminal bacteria stimulate immune cells underneath IECs.

Recent single cell analysis of IECs has uncovered heterogeneous gene expression, even in each subset of IECs (22). As discussed in this review, IECs are located at the interface between the luminal environment and host immune cells, which are stimulated by both of them. In addition, IECs transmit inside-out signals to luminal bacteria and outside-in signals to immune cells. This bidirectional stimulation of IECs may be one reason that each subset of IECs displays complicated gene expression patterns. In particular, specific commensal bacteria colonize their appropriate areas of the intestines and modulate epithelial physiology and immune responses. The gnotobiote system *in vivo* and development of commensal bacteria and organoid coculture and/or organ culture systems *in vitro* may provide useful information regarding how IECs respond to these luminal bacteria. Identification of these mechanisms is essential to better understand the host-microbiota interface and functional diversity of intestinal ECs. Furthermore, understanding of the role of IECs as a transmitter of luminal and immune signals is important for development of strategies to prevent bowel diseases including IBD, colonic cancer, and infection.

## Author Contributions

All authors listed have made a substantial, direct and intellectual contribution to the work, and approved it for publication.

### Conflict of Interest Statement

The author declares that the research was conducted in the absence of any commercial or financial relationships that could be construed as a potential conflict of interest. The reviewer KK is currently co-organizing a Research Topic with one of the authors YG, and confirms the absence of any other collaboration.
